# Program for the Neuropsychological Stimulation of Cognition in Students: Impact, Effectiveness, and Transfer Effects on Student Cognitive Performance

**DOI:** 10.3389/fpsyg.2019.01784

**Published:** 2019-08-13

**Authors:** Caroline de Oliveira Cardoso, Alessandra Gotuzo Seabra, Cristiano Mauro Assis Gomes, Rochele Paz Fonseca

**Affiliations:** ^1^Department of Psychology, Feevale University, Novo Hamburgo, Brazil; ^2^Department of Psychology, Mackenzie Presbyterian University, São Paulo, Brazil; ^3^Department of Psychology, Universidade Federal de Minas Gerais, Belo Horizonte, Brazil; ^4^Department of Psychology, Pontifical Catholic University of Rio Grande do Sul, Porto Alegre, Brazil

**Keywords:** executive functions, cognitive development, cognitive stimulation, neuropsychological intervention, prevention

## Abstract

Despite the crucial role played by the executive functions (EF) to cognitive, emotional, and social development of children before and during school years, little attention has been given to construct and analyze the efficacy of programs that intend to develop them. The program of neuropsychological stimulation of cognition in students: emphasis on EF, or PENcE (an acronym from its original name in Portuguese, *Programa de Estimulação Neuropsicológica da Cognição em Escolares: ênfase nas Funções Executivas*), is an early and preventive intervention program for school-aged children, and implemented at school three times a week for 5 months. The PENcE was structured in four modules, each focusing on a different executive component: organization and planning, inhibitory control, working memory, and cognitive flexibility. The objectives of this study were to verify the effectiveness of the PENcE among elementary school children and to investigate whether there are transfer effects to other executive, cognitive, and academic abilities. The sample consisted of 113 children attending 3rd or 4th grade at two public elementary schools. Eight classes participated in the study, divided into two groups: an experimental group (EG) (four classes; *n* = 64), which received the intervention, and a control group (CG) (four classes; *n* = 49), which continued their regular school activities. The EF and academic skills of both participant groups were evaluated before and after the intervention. The EG showed significantly greater improvements in inhibitory control, working memory, and abstract planning relative to the CG, with a small to medium effect size. There were transfer effects to other cognitive and academic abilities. These findings suggest the PENcE may be a useful method of improving EF and could benefit both school-aged children and education professionals.

## Introduction

Studies show that both academic and professional success depend largely on executive functions (EF) (Carlson et al., 2004; Prince et al., 2007). These abilities help individuals regulate and control their thoughts, behaviors, and emotions, allowing them to match behaviors to goals. EF can be understood in terms of three interconnected skills: working memory (the ability to hold and mentally manipulate information in order to perform more than one task at a time), inhibitory control (the ability to resist impulses and control automatic responses), and cognitive flexibility (the ability to change perspective or attentional focus) (Miyake et al., 2000; Diamond, 2013). The interactions between these abilities contributes to the emergence of more complex or higher-level functions, such as reasoning, problem solving, and planning (Diamond, 2013).

Metacognition is a skill that is closely related to EF. It concerns, among other aspects, the understanding of one’s own knowledge and thoughts (Flavell, 1987; Dantas and Rodrigues, 2013). The development of EF begins very early [from 4 to 10 or 12 months of age, according to different authors (Diamond, 2013; Hendry et al., 2016)] and extends into adolescence or early adulthood (Best and Miller, 2010; Diamond, 2013). In fact, the development of these cognitive skills depends on both brain development and experience or environmental factors (Center on the Developing Child at Harvard University, 2011; Diamond, 2013). In Brazil, for example, in the current educational model, little instruction is given to students on how to enhance their EF. Most of the time, the school focuses on specific subjects, as well as competencies such as reading, writing, and mathematics. Even though students are increasingly required to complete projects, remember the dates of tests and assignments, and concentrate despite the presence of multiple distractions (Meltzer, 2010; Cardoso et al., 2016), they are hardly ever taught how to reflect about what they think and learn (metacognition) in a systematic way (Meltzer et al., 2007). Furthermore, students are seldom taught how to solve problems in a more flexible way (problem solving and mental flexibility) or control their impulses (inhibitory control). Many educators are interested in going beyond the school curriculum and providing opportunities for students to develop their cognitive abilities. However, they have no training or education on how to help students in this way. A study by León (2018) examined the content of pedagogy courses in the city of São Paulo, Brazil, and found that only 4.7% of institutions offered classes on the neurobiology or neuropsychology of learning. The result is an ever-widening gap between traditional teaching approaches and the real-world challenges facing students and teachers (Lopez-Rosenfeld et al., 2013).

To provide support to teachers and students and contribute to educational improvement, scientists, educators, and psychologists have become increasingly focused on understanding the methods and approaches that can be used to stimulate the development of EF in the school environment (Barnett et al., 2008; Bierman et al., 2008; Dias and Seabra, 2013). Studies show that early interventions to promote the development of such abilities can produce direct long-term benefits to school performance, minimizing academic difficulties and contributing to the reduction and prevention of social, and mental health issues (Bull et al., 2008; Thorell et al., 2009; Roebers et al., 2011).

The efficacy and effectiveness of existing programs aimed at stimulating the EF in children were analyzed in a recent systematic literature review, which examined 19 studies, mostly involving preschool students (Cardoso et al., 2016). The authors found that the interventions were successful in stimulating the EF of healthy children. The majority of programs described in the literature were computer based. Though the interventions led to improvements in targeted skills, the presence of transfer effects to other areas (cognitive abilities and everyday activities) has not been established. In addition to computer-based training, some studies have proposed a curriculum-integrated approach, with activities included in the regular school curriculum. This strategy seems to have broader and more generalizable effects, since it stimulates several cognitive components simultaneously, and tends to be more intensive than interventions outside the classroom setting. However, the review also identified significant heterogeneity in sample characteristics and in the instruments used for pre- and post-intervention assessments, which interfered with the comparison of results across studies (Cardoso et al., 2016). In the school setting, the following programs stand out: Tools of Mind (Bodrova and Leong, 2007; Diamond et al., 2007; Barnett et al., 2008); PATHS (Promoting Alternative Thinking Strategies) (Riggs et al., 2006); and the Program of Intervention and Self-Regulation and EF, or PIAFEx, an acronym for its original name in Portuguese (*Programa de Intervenção em Autorregulação e Funções Executivas)* (Dias and Seabra, 2013). The Tools of Mind is an American program based on Vygosky’s theory of cognitive development. It was designed to be implemented as part of the school curriculum in early childhood education and instructs teachers on how to assist in the promotion of students’ EF as part of their everyday practice. The program involves 40 activities designed to promote sociodramatic play, encourage the use of private speech, and teach students to use external resources to stimulate self-regulation (Bodrova and Leong, 2007). The PATHS program focuses primarily on emotional and social skills in school-age children and was developed in the United Kingdom. The program was designed as a preventive intervention to be used by educators in the classroom. The PATHS provides teachers with the materials and instructions to teach children about emotions, self-control, social competence, and interpersonal problem-solving skills (Riggs et al., 2006). Last, the PIAFEx is a Brazilian program focused on the stimulation of EF in early childhood education and the 1st year of elementary school. It contains 43 activities and is implemented by teachers in the classroom settings. The activities are divided into 10 basic modules and one complementary section. The activities combine physical/motor activities, rule-based games, strategy learning, organization, as well as time, and goal management with the aim of enhancing the EF (Dias and Seabra, 2013). These interventions have led to improvements in cognitive flexibility and working memory (Diamond et al., 2007), inhibitory control (Röthlisberger et al., 2012; Dias and Seabra, 2015a, b), and social behavior (Barnett et al., 2008). However, most of these investigations focused on preschoolers or first-year elementary students. Very few programs have been developed for children in second grade and beyond (e.g., Rosário et al., 2007). In several countries, children are guaranteed the right to attend kindergarten, and preschool. However, in some South American countries such as Brazil, approximately 20% of children are not enrolled in preschool, despite having the right to do so (Brasil, 2013). This means that many children do not have the opportunity to enhance their executive functioning and benefit from interventions carried out before elementary school (Dias and Seabra, 2016).

In light of this shortcoming, a new program has been developed to stimulate EF of school-aged children, entitled Program for the Neuropsychological Stimulation of Cognition in Students: emphasis on EF, or PENcE (an acronym from its original name in Portuguese, *Programa de Estimulação Neuropsicológica da Cognição em Escolares: ênfase nas Funções Executivas*) (Cardoso and Fonseca, 2016). The program seeks to potentiate and optimize the development of EF and related cognitive processes through play, cognitive activities, and teaching of systematic strategies in the school setting. The purpose of this study was to investigate the efficacy of PENcE in elementary school students (grades 3–4). Furthermore, we sought to verify whether the program would have any transfer effects to academic performance, other cognitive components, and behavior. For this, different measures were used to investigate several components of EF.

## Materials and Methods

### Participants

The initial sample for the present study consisted of *n* = 160 elementary students in grades 3 and 4, recruited from two public schools in the city of Porto Alegre, Brazil. Eight teachers also took part in the study. The schools were chosen by convenience after their principals agreed to participate in the study. Both schools are in the same geographical area within the city of Porto Alegre, Brazil. The choice of two nearby schools was made in an attempt to control for demographic and socioeconomic variables. Since children in the experimental and control groups (CGs) attended the same schools, students and teachers were instructed not to discuss the intervention with one another. The importance of maintaining the confidentiality of intervention sessions was discussed with every participating teacher, who was also specifically instructed not to share or discuss with their colleagues any of the materials used in the intervention. Written informed consent was obtained from all parents, and assent forms were signed by every participating student.

The following exclusion criteria were applied: intellectual disability (25th percentile or lower on the Raven colored matrices test – Raven, 1938; adapted to Brazilian Portuguese by Angelini et al., 1999) (*n* = 13 children excluded); uncorrected sensory impairment (*n* = 0); genetic, psychiatric or neurological medical conditions (as reported by parents and teachers) (*n* = 10: *n* = 1 cerebral palsy, *n* = 1 major depression, *n* = 1 bipolar disorder, *n* = 7 attention deficit hyperactivity disorder); school absence rates of 25% or more during the execution of the program (*n* = 6); and age over 11 years and 11 months (*n* = 6). An additional *n* = 11 children transferred schools during the study and did not participate in the postintervention assessment, and *n* = 1 child was expelled from school during the postintervention assessment. Therefore, a total of *n* = 47 children were excluded from this study. Table 1 shows the characteristics of the children in each classroom, the condition to which they were assigned, the number of students with parental consent to participate, the number of participants excluded, and the final sample.

**TABLE 1:** Characterization of the participants in each classroom, the condition to which they were assigned, the number of students with parental consent to participate, the number of participants excluded, and the final sample.

**School**	**Class**	**Group**	**Children enrolled**	**Children with parental consent to participate in the study (%)**	**Children unable to participate in the study (%), considering the exclusion criteria**	**Final sample**
School 1	3°-32	EG	25	24 (96)	5 (20.8)	19
	3°-33	CG	18	15 (83.3)	4 (26.6)	11
	4°-41	EG	23	21 (91.3)	6 (28.5)	15
	4°-42	CG	26	23 (88.5)	10 (43.4)	13
School 2	3°-A	CG	27	18 (66.6)	5 (27.7)	13
	3°-B	EG	29	22 (75.9)	4 (18.1)	18
	4°-A	EG	23	18 (78.3)	6 (33.3)	12
	4°-B	CG	23	19 (82.60)	7 (36.8)	12
Total				160 (82.5)	47 (29.3)	113

As shown in Table 1, the vast majority (82.5%) of children agreed to participate in the study. The number of children excluded and the final sample size were similar across different schools. Cluster random sampling was used to assign each class to a different condition, so that some classrooms were randomly allocated to the experimental group (EG) and others to the CG. Children and teachers in the EG participated in the PENcE, while children in the CG continued their regular school curriculum. Two classes in each school were assigned to the EG (one in 3rd grade and one in 4th grade), while two were assigned to the CG (one in 3rd grade and one in 4th grade), for a total of 194 children (EG *n* = 103; CG *n* = 91). A total of 160 children (82.4%) received parental consent to participate in this study (EG *n* = 85, corresponding to 82.5%; CG *n* = 75, corresponding to 82.4%). A total of 24.7% of children in the EG (*n* = 21) and 36% in the CG (*n* = 26) were excluded from participation. As such, the final sample consisted of *n* = 64 children in the EG and *n* = 49 in the CG. The sociodemographic characteristics of the final sample are shown in Table 2. A total of eight public school teachers participated in the study, all of whom were female. Though all had a background in pedagogy, only five had postgraduate degrees (EG *n* = 3; CG *n* = 2). The average age of teachers in the EG was 41.25 years (*SD* = 8.26), while the average age in the CG was 46.25 years (*SD* = 7.41). Those in the EG had been working as teachers for 15 years on average (*SD* = 8.99), whereas those in the CG worked for a mean of 16.75 years (*SD* = 8.54). Teachers in the EG had been in their current jobs for an average of 8.50 years (*SD* = 3.87), and those in the control condition for 7.00 years (*SD* = 5.83). Teachers in both groups rated their professional performance as good to very good.

**TABLE 2:** Characteristics of the final sample.

**Sample characteristics**		**EG *(n* = 64)**	**CG (*n* = 49)**	
		**M (SD)**	**M (SD)**	***p*-value**
Age		8.64 (0.70)	8.86 (0.84)	0.138^a^
Age at school entry		5.80 (0.98)	6.02 (0.91)	0.238^a^
Socioeconomic score		19.12 (4.99)	20.23 (5.23)	0.275^a^
		f (%)	f (%)	
Gender	Male	31 (48.4%)	21 (42.9%)	0.555^b^
	Female	33 (51.6%)	28 (57.1%)	
Preschool	Yes	51 (82.3%)	38 (79.2%)	0.682^b^
	No	11 (17.7%)	10 (20.8%)	
Grade repetition	Yes	05 (7.8%)	08 (16.7%)	0.169^b^
	No	59 (92.2%)	39 (81.3%)	
Maternal educational level Illiterate		01 (1.6%)	01 (2.0%)	0.166^b^
Basic education		13 (20.3%)	20 (40.8%)	
High school		36 (56.3%)	22 (44.9%)	
College		09 (14.1%)	03 (6.1%)	
No information		05 (7.8%)	03 (6.1%)	
Paternal educational level Illiterate		00 (0%)	01 (2.0%)	0.093^b^
Basic education		22 (34.4%)	24 (49.0%)	
High school		27 (42.2%)	09 (18.4%)	
College		07 (10.9%)	07 (14.3%)	
No information		08 (12.5%)	08 (16.3%)	

*^*a*^Variables compared between groups using Student’s *t*-tests. ^*b*^Variables compared between groups using chi-square tests.*

### Materials

The PENcE was structured in four modules, each focusing on a different executive component: (1) organization and planning, (2) inhibitory control, (3) working memory, and (4) cognitive flexibility. To make the program more engaging and fun, students watched the movie *A Bug’s Life*. From that point onward, every module in the program was presented by a different “ant” character. The “ants” and the “League of Mind Training Ants” were developed to accompany the program and improve the learning and consolidation of EF strategies. “Ant Beatrix” presents module 1 – organization and planning; “Ant Pedro” presents module 2 – inhibitory control; “Ant Patrícia” presents module 3 – working memory; and “Ant Fabio” presents module 4 – cognitive flexibility. The “League of Mind Training Ants,” on the other hand, was introduced as a group of more experienced ants who are called in to help whenever the other ants need assistance. Throughout the program, the “League of Ants” teaches children the strategies and activities used to help the other ants, encouraging them to learn, and participate in the activities. In addition to the games and activities offered in the program, teachers are encouraged to integrate EF strategies into school activities in subjects such as mathematics, Portuguese, and Science. Each module, in turn, was divided into three stages:

Stage 1 – Strategy: Psychoeducation and modeling. Students are taught what, where, when, and how to use a strategy associated with EF discussed in that module. After explaining each strategy, the teacher provided examples and activities to illustrate how and when it could be implemented, in addition to modeling the strategies themselves.
Stage 2 – Learning and strategy consolidation. In the second stage, students are encouraged to actively implement the strategies through games, cognitive tasks, and school activities. Participants in the program completed a total of 38 cognitive activities.
Stage 3 – Reflection and transfer to daily life and school activities. The teacher encouraged students to reflect on how they could apply what they had learned to everyday situations and school activities. While these ideas are presented, the teacher encourages discussion and reflection, providing feedback to students throughout the process.

As an example, an activity developed in each module will be presented. Additional details on the names and procedures of different tasks in the PENcE are available in Appendix 1, as well as the recent book by Cardoso and Fonseca (2016).

#### Module 1: Organization and Planning, Activities: Looking for the Diamond

In this task, students must find a way to get to a diamond on a game board. In addition to the board, each pair of students receives a puppet, which they must place in a specific location. Then, the students are asked to find the best way to get the puppet to the diamond. Before making any moves, they must write down the planned path on a piece of paper using arrows to show the direction of each movement and stating the number of total steps required to get to the diamond. Only then can they move the puppet as planned.

#### Module 2: Inhibitory Control, Activities: Opposites Game

In this activity, the students must first name a series of pictures shown by their teacher. Then, they are shown a second set of pictures but are asked to say the opposite of what each picture shows.

#### Module 3: Working Memory, Activities: Sequencing

In this activity, students are shown a sequence of pictures of fruit and stationery. After the students see the sequence, the pictures are shuffled, and students are asked to put them back in their original order, organizing the fruits first and then the stationery items.

#### Module 4: Cognitive Flexibility, Activities: A New Ending for the Movie

After watching a movie, the teacher asks the students to imagine different endings for the film, and encouraging them to consider new possibilities and think in different ways. The teacher writes down all the ideas on the blackboard.

### Procedures

This study was conducted as part of a larger project, approved by the Ethics Committee of the Pontifical Catholic University of Rio Grande do Sul (PUCRS) (project number 1.035.498). After the study was approved by the committee, we contacted the schools and requested authorization to carry out the investigation. Informed consent was obtained from the legal guardians of all participants prior to the beginning of the study. During the preintervention assessment, the children signed a Term of Assent. The study was conducted in three stages: (1) preintervention assessment, (2) program implementation, and (3) postintervention assessment. Each stage is explained in detail below. The assessments and the intervention were performed during the school year, in the school setting, in bright and airy rooms.

#### STAGE 1: Preintervention Neuropsychological Assessment

All students underwent clinical and neuropsychological evaluations prior to the intervention. All instruments were administered individually, except for the “single word writing test” and the “arithmetic subtest of the school achievement test (SAT), 4th grade,” which were administered collectively. The assessment took place in a suitable setting, during regular school hours, with sessions lasting approximately 1 h and 30 min.

#### STAGE 2: Program Implementation

Prior to the intervention, each teacher in the EG took part in three individual training sessions. The teachers received printed material with key information on the PENcE and a proposed implementation schedule. Teachers also had weekly meetings with one of the cotherapists participating in the program, where they would discuss the activities performed as part of the intervention, clarify any questions, and present the tasks for the following week. These meetings lasted for 20–30 min and happened during school breaks or whenever the children were involved in activities outside the classroom.

The program was implemented by two neuropsychologists and a teacher from the EG. Group sessions were carried out in the children’s regular classroom, three times a week for 5 months. Each session lasted approximately 50–60 min. The teachers conducted the program activities while the neuropsychologists acted as cotherapists, assisting the teachers and participating in two of the weekly intervention sessions The cotherapists were two neuropsychologists who split their time between the four classrooms of the EG according to a predetermined schedule (for more information, see Cardoso and Fonseca, 2016).

#### STAGE 3: Postintervention Assessment

Shortly after the completion of the program, clinical, and neuropsychological assessments were carried out for all students, using the same instruments as the preintervention assessment. The evaluation was performed by members of the research group who did not participate in the intervention and were blind to participant group.

### Instruments Used in Pre- and Postintervention Assessments

Questionnaires Answered by Parents or Guardians:

(1) Sociodemographic and health questionnaires. These instruments were used to screen for medical issues and investigate the child’s developmental history, as well as parental education levels (socioeconomic status). This questionnaire was given to parents so they could provide additional information about their children, including age, education level, date of birth, socioeconomic status, history of grade retention, diagnosed physical or psychological illnesses, current medication use, previous hospitalizations, and current medical treatments. This information was used for sample characterization purposes.

#### Assessment of Intellectual Functioning

Cognitive functioning was evaluated using several different instruments in order to evaluate all the components of the EF. All the instruments used have been adapted for the Brazilian population, and national normative data are available in all cases. In addition, all instruments have evidence of validity and reliability.

(1) Raven colored matrices test [Raven, 1938; adapted to Brazilian Portuguese by Angelini et al. (1999)]. This is a measure of non-verbal and fluid intelligence. It contains 36 items divided into three groups of 12 items each, distributed in ascending order of difficulty. Correct answers are summed to provide a total score (range of scores: 0 – 36 points).

#### Assessment of EF

(1) Hayling test [(Burgess and Shallice, 1997; Fonseca et al., 2010); adapted for use in Brazilian children by Siqueira et al. (2016)]. This instrument evaluates the following executive components: inhibition, initiation, cognitive flexibility, and processing speed. The test contains 20 sentences, divided into two parts (A and B) of 10 sentences each. In part A, the child must complete each sentence with a context-compatible word. In part B, the child must complete the sentences with a word that is not related to the general meaning of the statement. The variables measured are total reaction time until an answer is given (total time part A and total time part B), number of errors (total errors part A and total errors part B/10) (range of scores: 0 – 10 points), number of errors in part B divided by 30 (total errors part B/30) (range of scores: 0 – 30 points), and ratio of the time taken to complete parts A and B (time B/time A).
(2) Go/No-Go Task from the Child Brief Neuropsychological Assessment Battery NEUPSILIN-Inf (Salles et al., 2016). This instrument primarily measures cognitive inhibition. An audio recording with 60 random numbers is played. The child is instructed to say “yes” every time he or she hears a number, but keeps silent when he or she hears the number 8. The total number of correct answers and omission and commission errors was calculated for each child (range of scores: 0 – 60 points).
(3) Unconstrained, letter, and category fluency (Jacobsen et al., 2016). These task involve several executive components, as well as lexical and semantic memory and linguistic abilities. In the unconstrained fluency test, the child is given 2 min and 30 s to say as many words as possible. In the letter fluency test, the child is given 2 min to elicit words that begin with the letter “p.” Last, in the category fluency test, the child is given an additional 2 min to name as many clothing items as they can. The total number of correct responses was obtained for each fluency test.
(4) Digit Span Subtest from the Wechsler Intelligence Scale, 3rd version [(Wechsler, 1999), adapted by Figueiredo, 2002]. This subtest examines attention and auditory working memory. Numbers are presented in direct and reverse orders. In the first case, also known as forward digit span, the child must repeat the numbers listed by the examiner in the same order they were presented. The backward digit span portion is similar to the first, except the child must repeat the numbers in reverse order of presentation. The number of correct responses on the forward and backward digit span tasks was calculated separately, and then added up to yield a total score. With each hit, the child gets a point: the forward digit span tasks range of scores, 0 – 16 points; the forward and backward digit span tasks range of scores, 0 – 14 points; total score range of scores, 0 – 30 points.
(5) Wisconsin card sorting test (WCST)–short version (Kongs et al., 2000). This test evaluates reasoning, abstract planning, cognitive flexibility, and rule maintenance. The task has 64 cards, which the participant must match to four stimulus cards, according to a rule set by the examiner. The children are given feedback on their response after every attempt. The cards can be matched by either color, shape, or number. The variables collected for this test were the total number of trials (maximum score 64), number of completed categories (range of scores: 0 – 3), number of errors (range of scores: 0 – 64), number of perseverative errors (range of scores: 0 – 63), and failure to maintain set.

#### Discourse Analysis

(1) Oral Narrative Discourse (OND) (Prando et al., 2016). In this test, the children must retell a story they were previously presented. The task has three stages: (a) partial retelling of the story; (b) complete retelling of the story; and (c) comprehension assessment, including 11 questions about the text. Throughout the test, the examiner must also determine whether the participant has understood the moral of the story. In this activity, the following scores were calculated: number of essential information items included in the partial retelling (range of scores: 0 – 18 points), total number of information items in the complete retelling (range of scores: 0 – 13 points), and comprehension (range of scores: 0 – 11 points).

#### Evaluation of Strategies and Academic Performance

(1) Arithmetic Subtest of the SATs (Viapiana et al., 2016a). This test evaluates basic arithmetic skills. Items are arranged according to their level of difficulty. Two different versions were used in the present study: one for 3rd grade children and another for 4th grade children. The overall number of correct responses was calculated for each child (3th grade children: range of scores, 0 – 47 points; 4th grade children: range of scores, 0 – 54 points).
(2) Single word writing test (Smythe and Everatt, 2000; Capovilla et al., 2001). Two single word writing tasks were used, one of which was drawn from the International dyslexia test (IDT). The instrument consists of a dictation test with 40 stimuli: 30 real words and 10 pseudowords. The examiner reads each word alone, then uses it in a sentence, and repeats the word again. The number of correctly written words (total: range of scores, 0 – 40 points; real words: range of scores, 0 – 30 points; pseudowords: range of scores, 0 – 10 points) was calculated for every participant.
(3) Decoding of words and pseudowords [developed by Moojen and Costa (2007)]. This task consists of a list of 40 regular and irregular words, plus 10 pseudowords. Through quantitative (number of correct and incorrect responses: range of scores, 0 – 50 points) and qualitative (types of errors) analyses, we identified the reading routes used to complete the task.

### Data Analysis

Data were analyzed descriptively and inferentially. Sociodemographic characteristics were compared between groups using chi-square and Student’s *t*-tests for categorical and continuous variables, respectively. We then tested for significant differences between the EG and CG on the preintervention assessment using Student’s *t*-tests. The presence and magnitude of intervention effects were determined using effect sizes (*d*), calculated as follows: d = Δ1 −Δ2/S_pooled_, where Δj = Xpos – Xpre and S_pooled_ = [QSIImage]. These analyses were conducted using the Wilson’s effect size calculator. We first computed the difference between group means for pre- and postintervention assessments (Δ1 and Δ2), before pooling the standard deviations of the four scores (EG pre- and postintervention, and CG pre- and postintervention). The correlation between measures in each group was also included in the calculation (Wilson, 2016). Lastly, the efficacy of the intervention was evaluated based on change scores, calculated as the difference between post- and preintervention assessments. Effect sizes were interpreted as described by Cohen (1988), with *d* = 0.20 suggesting a small effect, d near 0.50 representing a medium-sized effect, and d>0.80 indicating a large effect size. A 95% confidence interval (CI) was also calculated for each effect size. Lastly, after calculating the difference between the mean values of each variable, a *t*-test for independent samples was used to analyze whether these values differed between participant groups. Results were considered significant at *p* ≤ 0.05.

## Results

### Group Comparisons of Preintervention Results (Baseline)

In Table 2 the sociodemographic characteristics of the final sample are shown.

The groups did not differ with regard to gender, age, or socioeconomic status. The mean socioeconomic status of both groups was classified as C1 (low socioeconomic status, scores 23 – 28, mean family income of R$ 2,409.01/month), according to the Brazilian Criteria for Economic Classification, developed by the Brazilian Association of Research Companies (ABEP, 2014). Table 3 shows the comparison between the two groups (experimental vs. control) on the preintervention assessment. The groups did not significantly differ on any cognitive or behavioral measures.

**TABLE 3:** Comparison of preintervention assessment results between the experimental and CGs.

**Variables**	**EG M (SD)**	**CG M (SD)**	***p***
Raven – total correct responses	23.32 (4.23)	23.59 (4.96)	0.753
Verbal fluency – correct responses UVF	26.57 (12.21)	24.60 (14.19)	0.406
Verbal fluency – correct responses CVF	9.30 (4.82)	9.91 (3.80)	0.376
Verbal fluency – correct responses LVF	10.98 (4.22)	10.85 (4.02)	0.790
Go/No-Go – total correct responses	52.29 (5.70)	51.70 (5.93)	0.593
Go/No-Go – total omissions	4.30 (4.78)	4.75 (4.99)	0.619
Go/No-Go – total number of errors	3.46 (2.56)	3.52 (3.57)	0.937
OND – partial retelling EI	9.97 (4.25)	10.12 (4.02)	0.942
OND – partial retelling PI	12.54 (5.39)	15.66 (3.84)	0.889
OND – complete retelling	6.77 (3.83)	7.27 (3.22)	0.457
OND – text comprehension	8.03 (2.42)	7.71 (2.38)	0.419
Hayling test – total time part A	27.05 (15.68)	24.34 (14.29)	0.414
Hayling test – total errors part A	0.65 (1.03)	0.68 (0.88)	0.660
Hayling test – total time part B	48.33 (23.11)	40.49 (24.38)	0.565
Hayling test – total errors part B/10	5.78 (2.36)	5.37 (2.27)	0.563
Hayling test – total errors part B/30	14.97 (7.61)	13.72 (6.49)	0.595
Hayling test – time B/time A	2.04 (1.08)	2.05 (1.21)	0.885
Digit span fwd WISC-III	6.50 (1.46)	6.25 (1.61)	0.471
Digit span bwd WISC-III	3.29 (1.20)	2.91 (1.39)	0.092
Digits span fwd + Bwd WISC-III	9.81 (2.22)	9.18 (2.35)	0.152
Digit span fwd - Bwd WISC-III	3.21 (1.49)	3.33 (1.89)	0.521
WCST – number of trials	60.06 (7.43)	59.27 (7.51)	0.547
WCST – total errors	28.20 (12.35)	25.93 (13.76)	0.345
WCST – total perseverative errors	16.45 (11.66)	17.79 (13.39)	0.624
WCST – number of categories	1.85 (1.04)	1.96 (1.30)	0.760
WCST – conceptual-level responses	24.19 (11.69)	24.94 (12.96)	0.575
WCST – trials: First category	26.90 (20.31)	26.29 (19.67)	0.797
WCST – failure to maintain set	0.31 (0.65)	0.50 (0.85)	0.732
Arithmetic subtest–3rd grade	22.79 (5.78)	20.13 (5.89)	0.202
Arithmetic subtest–4th grade	17.27 (6.94)	16.31 (4.21)	0.260
Single word writing test – total correct responses	23.82 (6.34)	22.00 (7.58)	0.214
Single word writing test – total correct responses: pseudowords	5.42 (1.74)	4.95 (2.09)	0.279
Single word writing test – total correct responses: real words	18.40 (5.24)	17.04 (5.86)	0.232
Decoding – total correct responses	43.53 (8.73)	44.15 (6.69)	0.654
Decoding – real words	35.35 (7.20)	37.53 (3.53)	0.532
Decoding – pseudowords	8.18 (2.28)	8.42 (1.83)	0.863

*Raven, Raven’s colored progressive matrices; UVL, unconstrained verbal fluency; LVF, letter fluency; CVF, category fluency; EI, essential information; Fwd, forward; Bwd, backward; M, mean; SD, standard deviation.*

### Cognitive Measures: Group Comparison of Differences Between Post- and Preintervention Assessments

Table 4 presents the mean and standard deviation for each cognitive measure, as analyzed before and after the intervention. Effect sizes (d), CIs, and *p* values are also shown. The number of participants is not the same across all tests. This occurred because some children and parents refused to complete certain instruments and, in a few cases, because some participants were unable to complete some of the tasks.

**TABLE 4:** Cognitive measures: descriptive and inferential data regarding the comparisons between preintervention, postintervention, and change scores for the EG and CG.

**Variables**	**Group**	***N***	**M pre-**	**SD pre-**	**M post-**	**SD post-**	***r***	**Difference of means**	***d***	**CI**	***p***
**Raven’s colored progressive matrices**											
Total correct responses	EG	62	23.26	4.23	26.15	3.75	0.44^*^	2.89	**0.39**	**0.04/0.74**	**0.038**
	CG	48	23.75	4.88	25.04	3.60	0.69^*^	1.29			
**Go/No-Go**											
Total correct responses	EG	62	52.29	5.70	56.85	2.81	0.43^∗∗^	4.56	**0.46**	**0.03/0.89**	**0.044**
	CG	48	51.70	5.93	54.10	3.92	0.31^*^	2.40			
Omission errors	EG	62	4.30	4.78	1.67	2.24	0.34^∗∗^	2.63	0.33	−0.11/0.77	0.163
	CG	48	4.75	4.99	3.41	3.20	0.29^*^	1.34			
Commission errors	EG	62	3.46	2.56	1.41	1.24	0.11	2.05	0.40	−0.08/0.90	0.118
	CG	48	3.52	3.57	2.47	2.05	0.20	1.05			
**Hayling test**											
Time part A	EG	59	27.05	15.68	16.50	8.02	0.32^∗∗^	10.55	**0.53**	**0.09/0.97**	**0.035**
	CG	46	24.34	14.29	20.85	14.80	0.40^∗∗^	3.49			
Errors part A	EG	60	0.65	1.03	0.62	0.82	0.28^*^	0.03	0.08	−0.35/0.52	0.656
	CG	47	0.68	0.88	0.55	0.93	0.38^∗∗^	0.13			
Time part B	EG	56	48.33	23.11	34.68	12.26	0.31^∗∗^	13.65	0.37	−0.07/0.82	0.352
	CG	41	40.49	24.38	34.06	15.62	0.45^∗∗^	6.43			
Errors part B/10	EG	60	5.78	2.36	4.80	1.81	0.40^∗∗^	0.98	0.30	−0.13/0.74	0.166
	CG	46	5.37	2.27	5.04	2.05	0.33^*^	0.33			
Errors part B/30	EG	59	14.97	7.61	10.33	5.01	0.44^∗∗^	4.64	**0.45**	**0.03/0.87**	**0.044**
	CG	46	13.72	6.49	11.85	5.05	0.40^∗∗^	1.87			
Time B/time A	EG	57	2.04	1.08	2.48	1.31	0.48^∗∗^	−0.44	−**0.41**	−**0.83/0.03**	**0.069**
	CG	41	2.05	1.21	2.01	1.11	0.40^∗∗^	0.04			
**Digits WISC-III**											
Digit span fwd	EG	62	6.50	1.46	7.48	1.70	0.48^∗∗^	0.98	**0.28**	**0.03/0.74**	**0.042**
	CG	48	6.25	1.61	6.62	1.56	0.61^∗∗^	0.37			
Digits span bwd	EG	62	3.29	1.20	4.16	1.04	0.32^*^	0.87	0.25	−0.16/0.67	0.244
	CG	48	2.91	1.40	3.48	1.11	0.44^∗∗^	0.57			
Digit span fwd + bwd	EG	62	9.81	2.24	11.61	2.14	0.52^∗∗^	1.80	**0.41**	**0.06/0.76**	**0.034**
	CG	48	9.19	2.37	10.08	2.04	0.62^∗∗^	0.89			
Digit span fwd - bwd	EG	62	3.20	1.49	3.32	1.78	0.38^∗∗^	0.12	0.17	−0.23/0.59	0.410
	CG	48	3.33	1.89	3.14	1.76	0.45^∗∗^	−0.19			
Verbal fluency											
Total correct responses (UVF)	EG	60	26.57	12.21	33.00	11.33	0.54^∗∗^	6.43	0.01	−0.33/0.35	0.943
	CG	48	24.60	14.19	30.88	14.20	0.62^∗∗^	6.28			
Total correct responses (LVF)	EG	60	9.30	4.82	11.02	3.58	0.35^∗∗^	1.72	0.30	−0.11/0.72	0.161
	CG	47	9.91	3.80	10.36	4.30	0.42^∗∗^	0.45			
Total correct responses (CVF)	EG	60	10.98	4.22	12.18	3.87	0.46^∗∗^	1.20	0.07	−0.31/0.46	0.714
	CG	47	10.85	4.02	11.74	4.54	0.49^∗∗^	0.89			
**WCST**											
Number of trials	EG	60	60.06	7.43	53.10	10.73	0.41^∗∗^	6.96	0.15	−0.28/0.57	0.503
	CG	48	59.27	7.51	53.70	11.70	0.35^*^	5.57			
Total errors	EG	58	28.20	12.35	17.13	10.83	0.29^*^	11.07	**0.44**	**0.01/0.88**	**0.050**
	CG	46	25.93	13.76	20.22	11.49	0.43^∗∗^	5.71			
Perseverative errors	EG	58	16.45	11.66	8.61	6.68	0.20	7.84	0.04	-0.42/0.49	0.994
	CG	46	17.79	13.39	10.43	9.92	0.43^∗∗^	7.37			
Number of categories	EG	60	1.85	1.04	2.55	0.59	0.35^∗∗^	0.70	**0.48**	**0.08/0.88**	**0.020**
	CG	58	1.96	1.30	2.19	0.89	0.45^∗∗^	0.23			
Conceptual level	EG	60	24.19	11.69	31.68	5.92	0.18	7.49	0.42	−0.09/0.92	0.123
	CG	48	24.94	12.96	28.21	9.01	0.05	3.27			
Trials to complete first category	EG	60	26.90	20.31	16.17	10.24	0.36^∗∗^	10.73	0.22	−0.23/0.68	0.380
	CG	48	26.29	19.67	19.37	16.20	0.20	6.92			
FMS	EG	60	0.31	0.65	0.25	0.54	0.03	0.06	−0.18	−0.70/0.33	0.457
	CG	48	0.50	0.85	0.29	0.61	0.12	0.25			
**OND**											
Partial retelling EI	EG	61	9.97	4.25	13.38	3.02	0.48^∗∗^	3.41	0.09	−0.27/0.46	0.289
	CG	49	10.12	4.02	12.76	3.83	0.55^∗∗^	2.64			
Partial retelling PI	EG	61	12.54	5.39	15.66	3.84	0.50^∗∗^	3.12	0.22	−0.14/0.58	0.247
	CG	49	12.76	5.21	14.00	4.95	0.55^∗∗^	1.24			
Complete retelling	EG	57	6.77	3.83	8.86	2.34	0.49^∗∗^	2.09	**0.42**	**0.03/0.82**	**0.042**
	CG	49	7.27	3.22	8.04	2.87	0.47^∗∗^	0.77			
Comprehension	EG	60	8.03	2.42	9.10	1.92	0.58^∗∗^	1.07	0.14	−0.21/0.49	0.446
	CG	48	7.71	2.38	8.46	2.25	0.55^∗∗^	0.75			

Raven, Raven’s colored progressive matrices; UVL, unconstrained verbal fluency; LVF, letter fluency; CVF, category fluency; EI, essential information; Fwd, forward; Bwd, backward; M, mean; SD, standard deviation; FMS, failure to maintain set. Bold values - there was a significant difference between the groups in those items. The asterisks represent significant at ^*^p < 0.05 and ^∗∗^p < 0.001.

The groups differed significantly on some of the variables analyzed. Our measure of fluid reasoning (Raven’s Colored Progressive Matrices), for instance, showed a small to medium effect size between groups. Improvements in inhibitory control (total correct responses on the Go/No-Go test and total errors part B/30 on the Hayling test) were also significantly greater in the EG relative to the CG, with an effect size in the small to medium range. Students in the EG took more time to complete tasks involving inhibitory control relative to their counterparts in the CG. This finding suggests that participants in the EG were less impulsive and put more thought into complex tasks (time B/time A on the Hayling test) than children in the CG. Furthermore, a significant group effect was observed on a measure of initiation and processing speed (total time part A in the Hayling test), where the EG outperformed the CG with a moderate effect size. The EG also obtained higher scores than the CG on measures of auditory attention and short-term auditory memory (total correct responses–digit span forward and total score–digit span–WISC-III). However, the groups did not differ in terms of their working memory (total correct responses–digit span backward). Students in the EG also showed improvements on the complete retelling score of the OND task, which evaluates episodic memory, working memory, linguistic expression, synthetic reasoning, and planning. The results of the WCST also revealed greater improvements in the EG relative to the CG on abstract planning (number of completed categories and total number of errors). No group effects were observed on any of the verbal fluency tests, though postintervention scores did improve relative to the preintervention assessment.

### Measures of Academic Ability: Group Comparison of Differences Between Post- and Preintervention Assessments

Table 5 shows the results obtained by each group on measures of mathematical ability (Arithmetic Subtest for 3rd and 4th grades), reading (Decoding), and writing (single word writing).

**TABLE 5:** Measures of academic ability: descriptive and inferential data regarding of the comparisons between preintervention, postintervention, and change scores for the EG and CG.

**Variables**	**Group**	***N***	**M pre-**	**SD pre-**	**M post-**	**SD post-**	***r***	**Difference of means**	***d***	**CI**	***p***
**Arithmetic subtest of the SAT**											
SAT 3rd grade	EG	34	22.79	5.78	29.91	5.21	0.42^∗∗^	7.12	**0.57**	**0.04/1.10**	**0.041**
	CG	22	20.13	5.89	23.90	6.71	0.60^∗∗^	3.77			
SAT 4th grade	EG	22	17.27	6.94	23.59	6.34	0.40^*^	6.32	0.38	−0.21/0.99	0.950
	CG	23	16.31	4.21	20.21	6.40	0.55^∗∗^	3.90			
**Single word writing test**											
Total correct responses	EG	47	23.82	6.34	28.85	5.52	0.85^∗∗^	5.03	**0.45**	**0.22/0.69**	**<0.001**
	CG	41	22.00	7.58	23.92	7.96	0.86^∗∗^	1.92			
Pseudowords	EG	47	5.42	1.74	6.70	1.58	0.38^∗∗^	1.28	**0.78**	**0.36/1.19**	**<0.001**
	CG	41	4.95	2.09	4.70	2.44	0.69^∗∗^	−0.25			
Real words	EG	47	18.40	5.24	22.12	4.46	0.83^∗∗^	3.72	**0.26**	**0.09/0.52**	**0.046**
	CG	41	17.04	5.86	19.31	6.40	0.81^∗∗^	2.27			
**Word decoding**											
Total correct responses	EG	40	43.53	8.73	46.10	4.76	0.50^∗∗^	2.57	0.22	−0.22/0.66	0.363
	CG	33	44.15	6.69	45.27	5.01	0.58^∗∗^	1.12			
Real words	CG	40	35.35	7.20	37.53	3.53	0.55^∗∗^	2.18	0.18	−0.24/0.61	0.598
	EG	33	35.70	5.52	37.24	3.70	0.70^∗∗^	1.54			
Pseudowords	CG	40	8.18	2.28	8.58	1.77	0.49^*^	0.40	0.40	−0.06/0.86	0.195
	CG	33	8.42	1.83	8.03	1.98	0.52^∗∗^	−0.39			

M, mean; SD, standard deviation; d, effect size. Bold values - there was a significant difference between the groups in those items. The asterisks represent significant at ^*^p < 0.05 and ^∗∗^p < 0.001.

Children in the EG outperformed the CG on all variables obtained from the single word writing test. Among third graders, significant differences between the EG and CG were also observed on the arithmetic subtest from the SAT. Among fourth graders however, no differences were noted either in the SAT or the decoding test.

## Discussion

This study was designed to examine the effectiveness of an early preventive intervention to stimulate EF in the school setting. The program was aimed at school-aged children in elementary grades 3 and 4. First, we sought to verify whether children who completed the program showed improvements in executive abilities relative to CG counterparts. Afterward, we investigated the presence of transfer effects to other cognitive (e.g., attention, fluid reasoning, and processing speed) and academic abilities (mathematics, word reading, and single word writing).

Our findings revealed significant differences between the EG and CG on several measures of EF, with improvements in the following executive components: inhibitory control, abstract planning, and complex verbal working memory. The effect size of these differences ranged from low to moderate. The results are aligned with previous studies, which found that executive components can be improved by school-based interventions (Lizarraga et al., 2003; Diamond et al., 2007; Barnett et al., 2008; Röthlisberger et al., 2012; Dias and Seabra, 2015a, 2016; Traverso et al., 2015). Furthermore, we observed transfer effects to attention, fluid reasoning, academic abilities, and behavior. These and other benefits of the PENcE will be discussed in more detail below. It is important to note that outcomes were evaluated through both formal methods (i.e., performance tests) as well as functional and ecological measures.

With respect to inhibitory control, we found that children in the EG had better impulse control than their CG counterparts. This was deduced from the fact that children in the experimental condition obtained significantly better scores than children in the control condition on the following measures: Total correct responses on the Go/No-Go test and total number of errors/30 on the Hayling test. Previous studies have found similar results among preschoolers (Dias and Seabra, 2015a; Traverso et al., 2015) and first graders (Röthlisberger et al., 2012; Dias and Seabra, 2015b). Additionally, as suggested by a marginally significant group difference on time B/time A of the Hayling test, the EG appeared to take longer than the CG to respond to complex situations involving impulse control. At first glance, this result may create the false impression that participants exposed to the intervention have become less flexible. However, we believe this finding can be better explained by the fact that children in the EG may have attempted to improve their accuracy by slowing down task execution, which indicates a decrease in impulsivity. Dias and Seabra (2015a) also found that children who participated in a cognitive intervention program showed an increase in reaction time when responding to a complex situation, indicating they took some time to think before providing a response.

This study also showed that children in the EG demonstrated improvements in initiation and processing speed (as measured by total time on part A of the Hayling test). This result indicates an enhancement in automatic skills. In other words, in more automatic situations, participants in the EG were faster to respond. Therefore, the present findings suggest that school-based interventions can lead to improvements in inhibitory control, processing speed, and initiation in typically developing children. Although not all measures showed significant differences in the improvement of CG and EG participants over time, all significant differences favored the EG, that is, in no case did the CG present greater gains than the EG.

With regard to working memory, though the groups did not differ on the backward digit span (WISC-III), significant differences were identified on the complete retelling variable from the OND task. This may be attributed to improvements in episodic memory skills and complex verbal working memory in EG participants. Unlike the Backward Digit Span, the OND task evaluates cognition in a contextualized situation (narrative), which places additional demands on linguistic expression, synthetic reasoning, and planning. Indirectly, this task also recruits working memory processes, since the individual must retain the information from previous paragraphs and integrate it with more recent information in order to understand and retell the story. Previous studies involving school-based intervention programs have produced inconsistent findings in this regard, with some identifying significant group differences (Lizarraga et al., 2003) and others failing to do so (Röthlisberger et al., 2012; Dias and Seabra, 2015a, b). However, there is an important difference between these studies: the program developed by Lizarraga et al. (2003) was aimed at private school students with an average age of 13 years. The present investigation, along with other related studies (Röthlisberger et al., 2012; Dias and Seabra, 2015a, b) focused on preschoolers and early primary school children. The PENcE also appeared to have an impact on planning skills, as evidenced by moderate group differences on the number of completed categories on the WCST.

With respect to cognitive flexibility, although the EG showed improvements relative to its preintervention assessment, there were no differences between groups on the total number of perseverative errors on the WCST. Similar results were found by Dias and Seabra (2015a) in preschoolers and by Röthlisberger et al. (2012) in school-aged children. Other studies, however, found improvements in cognitive flexibility among participants in the EG (Lizarraga et al., 2003; Diamond et al., 2007; Dias and Seabra, 2015b). One possible explanation is the format of the tasks used to evaluate cognitive flexibility. Instruments such as the trial making test and Flanker test require that the individual respond to a set of stimuli in a given manner, until they are instructed by the examiner to change the type of response provided. Other tasks, like the WCST, also involve changing response patterns; however, in this case, the examiner does not explicitly inform the participant when they must change their response. The participant must infer the need to adopt a new response pattern based on feedback and observation, which requires significantly more abstract reasoning.

Another possible explanation for these findings is that attention and inhibition begin to develop in early childhood, while working memory and flexibility are more complex and begin to develop later (Karbach and Kray, 2009; Dawson and Guare, 2010; Diamond, 2013; Dias and Seabra, 2015a). It is also possible that these abilities can only be affected by longer interventions, as proposed in programs with an estimated duration of 1–2 years (Lizarraga et al., 2003; Diamond et al., 2007; Barnett et al., 2008). As such, we recommend that the working memory and cognitive flexibility modules be extended and complemented by additional activities. This must also be accompanied by an increase in the length of teacher training periods. We might also consider the structure of the program, in which the working memory and cognitive flexibility modules were the last to be presented. Thus, they are stimulated for less time than components introduced earlier in the program, such as planning and inhibitory control. Another hypothesis is that the benefits of the program may only become evident later (Diamond and Ling, 2015). Some authors have not identified improvements in EF immediately after an intervention but did identify group differences on follow-up assessments (Hermida et al., 2015; Dias and Seabra, 2016).

In addition to initiation and processing speed, there was evidence of transfer effects to attention and short-term memory, as measured by the Forward Digit Span (WISC-III). There also appeared to be transfer effects to fluid reasoning/intelligence, as measured by Raven’s colored progressive matrices. In the digit span forward, children from the EG showed greater gains than their CG counterparts. This result supports the hypothesis that EG participants showed improvements in automatic abilities. The relationship between fluid reasoning and EF has been widely researched, and many authors have identified a close association between these two constructs, especially during childhood (Friedman et al., 2006; Brydges et al., 2012; Diamond, 2013). Furthermore, in the past few years, several studies have shown that certain interventions on EF have positive impacts on fluid intelligence (Klingberg et al., 2002, 2005; Lizarraga et al., 2003; Jaeggi et al., 2008; Klingberg, 2010; Bergman Nutley et al., 2011). For instance, studies where a computer program was used to stimulate working memory in children with ADHD showed that participants also experienced significant gains in fluid intelligence, also measured by the Raven test (Klingberg et al., 2002, 2005). On the other hand, when this protocol was administered to healthy preschoolers in a different study, no improvements on the Wechsler Intelligence Scale for Preschoolers were identified in children exposed to the intervention (Thorell et al., 2009). Therefore, the literature is not unanimous with regard to the transfer effects of cognitive training programs to fluid intelligence.

The PENcE also had a positive impact on academic abilities, namely, mathematics and single word writing. There is a strong relationship between EF skills (especially working memory) and mathematical ability. In fact, some studies consider EF a predictor of academic ability (Blair and Razza, 2007; Raghubar et al., 2010; Cragg and Gilmore, 2014). Third graders in the present study showed significant improvements in mathematical ability relative to the CG, who maintained their regular school activities. However, these differences were not observed among fourth graders. The test is more demanding for third graders, which may be why the effects were more evident in this population (Viapiana et al., 2016b). In the single word writing test, the EG showed significantly better performance than the CG for both real words and pseudowords. Previous studies have also shown that interventions to stimulate EF in typically developing children can have a positive impact on academic skills such as reading (Loosli et al., 2012; Karbach et al., 2015), mathematics (Söderqvist and Bergman Nutley, 2015; Dias and Seabra, 2016), and writing (reduced errors in syntax and orthography) (Hooper et al., 2006). Despite improvements in some academic abilities, the EG did not show significant differences in word decoding or school performance (as graded by their teachers). In the study conducted by Rosário et al. (2010), the authors found that participants in the EG improved their knowledge of learning strategies but did not show significant improvements in school performance in mathematics or Portuguese.

Some limitations of the present study must be taken into consideration. This program worked exclusively with teachers and students and did not involve parents. Additionally, our CG did not actively participate in a cognitive training program. Moreover, the use of mixed outcome measures may have made it more challenging to discuss the present findings. We recommend that future studies work on adapting the PENcE to high school students and different clinical groups (e.g., children with ADHD or learning disorders), so that the program can be used in different contexts. The PENcE may also be implemented in the public schools of developing countries as a public policy initiative. Additionally, we recommend a follow-up study to evaluate whether the results change over time. The program may also be complemented by meetings with parents and guardians to raise awareness and provide guidance on how EF can be stimulated at home and in daily life activities. Parents’ understanding of executive functioning can help maintain the effects of the intervention in everyday life (Volckaert and Nöel, 2015). We also suggest that the program be amplified to include a module focused on “hot” EF and tasks used to evaluate them. “Hot” EF are related to emotional processes and include motivation, decision-making, emotion regulation, and responses to reward and punishment. “Cold” EF, on the other hand, are more closely related to logical and cognitive processes, such as logical and abstract reasoning, planning, problem solving, and working memory (Zelazo et al., 2005). The PENcE focused on cold EF, and as such, it may benefit from the inclusion of an additional module that deals with emotion regulation. Last, another limitation of this study is that systematic assessments of fidelity were not carried out. Future studies may complement the measures used in the present investigation with records of the number of children who implemented the strategies and used them throughout the intervention (performance records) or in their daily lives.

This study demonstrated the efficacy of the PENcE and showed that it is possible to stimulate EF in school settings with an early preventive intervention for children in elementary school grades 3 and 4, even in poor socioeconomic conditions. Children who participated in the EG outperformed their CG counterparts in several outcome measures. We believe that the ecological setting of the program, the use of a children’s story to set up the intervention, and the inclusion of cognitive activities and games were highly motivational, improving engagement and the establishment of mnemonic, and emotional connections among participants. This structure provided the opportunity for exploration, active learning, and the use of visual stimuli, all of which are known to be beneficial for students (Marzano, 2003). Children seldom refused to participate in the program or specific activities. Another positive aspect of the intervention was that it took place in children’s school, complementing the regular curriculum. This program was unique in the sense that teachers were encouraged to include EF strategies in other classroom activities, which may have increased transfer effects. Finally, this program encouraged the use of explicit and systematic strategies, as well as reflection, which plays a fundamental role in the development of EF (Espinet et al., 2013).

## Data Availability

All datasets generated for this study are included in the manuscript and/or the supplementary files.

## Ethics Statement

This study was conducted as part of a larger project, approved by the Ethics Committee of the Pontifical Catholic University of Rio Grande do Sul (PUCRS) (Project No. 1.035.498). After the study was approved by the committee, we contacted the schools and requested authorization to carry out the investigation. Informed consent was obtained from the legal guardians of all participants prior to the beginning of the study.

## Author Contributions

CdC wrote the manuscript, analyzed the data, and implemented the intervention in schools. AS assisted in the writing of the manuscript, and reviewed the results and discussion. CG made and assisted the statistical analyses. RF guided the study and reviewed the entire manuscript.

## Conflict of Interest Statement

The authors declare that the research was conducted in the absence of any commercial or financial relationships that could be construed as a potential conflict of interest.

## Appendix

Structure of the PENcE (Cardoso and Fonseca, 2016)

**Table:**

**INTRODUCTION**
Presentation of the program and movie (“Bug’s Life”)
**MODULE 1: ORGANIZATION AND PLANNING**
Strategy: Three steps: planning (taking the time to think before starting a task); execution (thinking while doing the task); and assessment (reflect and assess whether the goals of the task were achieved)
**Stage 1: Strategy acquisition: Psychoeducation and modeling**
Psychoeducation: Introduction of “Ant Beatrix,” a ballerina who has trouble with planning, and gets confused when she has many things to do
Modeling: Activities: Packing your backpack and Creating a notebook cover
**Stage 2: Learning and strategy consolidation**
The following activities were developed as part of this section:
- Dot game
- Looking for the diamond
- Logical sequence
- Building a bug
- Cooking
- School activities
**Stage 3: Reflection and transfer to daily life and school activities**
Recap of the section and strategies learned; opportunity to discuss and reflect
**RECAP OF PREVIOUS MODULES**
Writing a text
**MODULE 2: INHIBITORY CONTROL**
Participants are taught the “Stop, Think, and then Go” strategy”
**Stage 1: Strategy acquisition: Psychoeducation and modeling**
Psychoeducation: Introduction of “Ant Pedro,” who loves playing soccer, but is very impulsive and has trouble waiting his turn
Modeling: Activities: Opposites game and looking for the target
**Stage 2: Learning and strategy consolidation**
The following activities were developed as part of this section:
- Dancing
- Looking for the target
- Willpower
- Simon says
- Card game: Snap
- School activities
**Stage 3: Reflection and transfer to daily life and school activities**
Recap of the section and strategies learned; opportunity to discuss and reflect
**RECAP OF PREVIOUS MODULES**
- Birthday party
- Building an object: a closed mouth catches no flies
**MODULE 3: WORKING MEMORY**
Strategy – Four steps are suggested: (1) paying attention to the stimulus/instruction; (2) memorizing new information – use of mental repetition and visualization; (3) mental organization of information; and (4) performing activities slowly, focusing on quality rather than speed
**Stage 1: Strategy acquisition: Psychoeducation and modeling**
Psychoeducation: Introduction of “Ant Patricia,” who loves fashion and wants to be a model; but is very forgetful, and can’t keep track of multiple items of information or instructions
Modeling; activities: Image sequencing; body parts; and numbering the sequence
**Stage 2: Learning and strategy consolidation**
The following activities were developed as part of this section:
- Sequencing
- Differences game
- The missing one
- Completing sentences
- Numbering the sequence
- School Activities
**Stage 3: Reflection and transfer to daily life and school activities**
Recap of the section and strategies learned; opportunity to discuss and reflect
**RECAP OF PREVIOUS MODULES**
- Following instructions
- Crazy sentences
**MODULE 4: COGNITIVE FLEXIBILITYS**
Strategy: When something unexpected happens or a problem arises, we need to think of multiple alternatives
**Stage 1: Strategy acquisition: Psychoeducation and modeling**
Psychoeducation: Introduction of “Ant Fabio,” who sings in a band with his classmates. When he comes across a new situation or his plans fall through, he has trouble thinking of different ways to resolve the situation
Modeling: activities: A new ending for the movie and group drawing
**Stage 2: Learning and strategy consolidation**
The following activities were developed as part of this section: - Taking a new perspective - Switching game - Matching cards - Crack the code - A new ending - School activities
**Stage 3: Reflection and transfer to daily life and school activities**
Recap of the section and strategies learned; opportunity to discuss and reflect
**RECAP OF PREVIOUS MODULES**
Complete all modules Activities:
- A new ending for the “Three Little Pigs”
- Building a different tower
- Picnic
